# Vagus nerve stimulation does not improve recovery of forelimb motor or somatosensory function in a model of neuropathic pain

**DOI:** 10.1038/s41598-022-13621-3

**Published:** 2022-06-11

**Authors:** Katherine S. Adcock, Tanya Danaphongse, Sarah Jacob, Harshini Rallapalli, Miranda Torres, Zainab Haider, Armin Seyedahmadi, Robert A. Morrison, Robert L. Rennaker, Michael P. Kilgard, Seth A. Hays

**Affiliations:** 1 The University of Texas at Dallas, Texas Biomedical Device Center, 800 West Campbell Road, Richardson, TX 75080-3021 USA; 2 School of Behavioral and Brain Sciences, The University of Texas at Dallas, 800 West Campbell Road, Richardson, TX 75080-3021 USA; 3 Department of Bioengineering, Erik Jonsson School of Engineering and Computer Science, The University of Texas at Dallas, 800 West Campbell Road, Richardson, TX 75080-3021 USA

**Keywords:** Regeneration and repair in the nervous system, Neurological disorders

## Abstract

Nerve injury affecting the upper limb is a leading cause of lifelong disability. Damage to the nerves in the arm often causes weakness and somatosensory dysfunction ranging from numbness to pain. Previous studies show that combining brief bursts of electrical vagus nerve stimulation (VNS) with motor or tactile rehabilitation can restore forelimb function after median and ulnar nerve injury, which causes hyposensitivity of the ventral forelimb. Here, we sought to determine whether this approach would be similarly effective in a model of radial nerve injury that produces allodynia in the ventral forelimb. To test this, rats underwent complete transection of the radial nerve proximal to the elbow followed by tubular repair. In the first experiment, beginning ten weeks after injury, rats received six weeks of tactile rehabilitation, consisting of mechanical stimulation of either the dorsal or ventral region of the forepaw in the injured limb, with or without concurrent VNS. In a second experiment, a separate cohort of rats underwent six weeks of forelimb motor rehabilitative training with or without paired VNS. Contrary to findings in previous models of hyposensitivity, VNS therapy fails to improve recovery of either somatosensory or motor function in the forelimb after radial nerve injury. These findings describe initial evidence that pain may limit the efficacy of VNS therapy and thus highlight a characteristic that should be considered in future studies that seek to develop this intervention.

## Introduction

Damage to the median, ulnar, or radial nerves in the upper limb is common and often debilitating^1,2^. Injuries to these nerves produce weakness, loss of coordination, and sensory dysfunction in the arm and hand^3,4^. Despite significant advances in surgical and rehabilitative treatments, few individuals regain full function. The development of therapeutic strategies to restore motor control and normalize somatosensation holds promise to reduce disability arising from these conditions.

A number of recent studies demonstrate that pairing short bursts of vagus nerve stimulation with motor and sensory rehabilitation promotes recovery after forelimb nerve injury^5–7^. This approach is premised on the ability of VNS to enhance synaptic plasticity in the central nervous system, which in turn supports recovery^5,8^. In these studies, lesion of the median and ulnar nerves produces chronic forelimb weakness and hyposensation of the ventral surface of the forepaw. Pairing VNS with motor rehabilitative training increases forelimb strength and significantly improves recovery of motor function^5^. Similarly, delivery of VNS during tactile rehabilitation reduces chronically elevated withdrawal thresholds and enhances recovery of forelimb somatosensory function^6,7^.

While these studies provide foundational evidence to support the potential utility of VNS therapy for nerve injury, they restrict focus to recovery of function in the context of hyposensation. Neuropathic pain, a common consequence of nerve injury, is not captured in these models. Thus, we sought to determine whether VNS therapy can similarly enhance recovery of forelimb function in a model of nerve injury that incorporates neuropathic pain. To do so, rats underwent transection and repair of the radial nerve in the proximal forelimb, which produces chronic allodynia and forelimb weakness^9^. In a first experiment, rats received VNS paired with tactile rehabilitation with or without concurrent VNS. Mechanical withdrawal thresholds in the injured forelimb were assessed over the course of therapy. In a second experiment, a separate cohort of rats received motor rehabilitation of the previously injured limb with or without concurrent VNS and forelimb strength was measured over the course of therapy. Contrary to findings after median and ulnar injury, we report that multiple implementations of VNS therapy failed to promote recovery of either forelimb somatosensation or motor function. Below, we describe these findings and explain the relevance of these results in refining future clinical applications of VNS-based therapies.

## Methods

### Subjects

Seventy-eight adult female Sprague–Dawley rats, each weighing approximately 250 g when they entered the study, were used. Sample size was selected based on previous studies employing a similar design^6,7^. All animals were housed in a 12:12 h reversed light–dark cycle and were food deprived during motor training. Twenty-five animals were excluded from the study based on pre-determined criteria: VNS device failure (n = 4), mortality (n = 7), failure to display a motor deficit after lesion, as defined by an average post-lesion baseline performance with at least 30% of trials exceeding 60 degrees on the supination task (n = 14). All protocols were approved by The University of Texas at Dallas Institutional Animal Care and Use Committee (Protocol #14–10). All experiments described here conform to the ARRIVE guidelines.

### Forepaw mechanical sensory testing

Forelimb mechanical withdraw thresholds were assessed before injury, 10 weeks after injury (‘After Injury’), and 16 weeks after injury (‘After Therapy’). Testing procedures were performed as described previously^6,7^. Assessment was performed in an acrylic chamber on a wire mesh floor. Mechanical sensitivity was tested on the right and left forepaws using a Dynamic Plantar Aesthesiometer (Catalog Number: 37550, Ugo Basile, Italy), which automatically detects and records force at the time of paw withdrawal^5,6,10^. The actuator filament (0.5 mm diameter) was placed on the plantar (ventral) surface of the forepaw, and a linearly increasing force was applied (20 s ramp time, 50 g maximal force). The force at which paw withdrawal occurred was recorded for analysis. The left and right paw were alternately tested, with a minimum 1 min interval between consecutive tests. The average force at withdrawal over 5 trials was calculated for each paw. Experimenters were blinded to group throughout assessment.

### Forelimb motor assessment

To assess skilled forelimb motor function, a subset of animals underwent training on the supination task, as previously described^11–13^. The behavioral training apparatus consisted of an acrylic cage with a slot through which animals reach, grasp, and supinate their forelimb to rotate a spherical manipulandum. The manipulandum was affixed to a rotary encoder to measure turn angle. If an animal rotated the manipulandum past a predetermined angle within 2 s of initiating contact, the trial was recorded as a success and a food reward was delivered to a hopper in the cage (45 mg dustless precision pellet, BioServ, Frenchtown, NJ). If the turn angle did not exceed the threshold within the 2 s, the trial was recorded as a failure and no food reward was given. Control software adaptively scaled the turn angle required to receive a reward for each trial based on the median of the preceding 10 trials to a maximum turn angle threshold of 60°.

Training sessions occurred twice a day for 30 min each, 5 days a week. Pre-injury training continued until animals achieved a 75% success rate, defined as trials in which the turn angle exceeded 60°, averaged across 6 consecutive training sessions. Data from these six sessions is reported as the ‘Before Injury’ time point. All animals then received a radial nerve injury, according to the procedures described below. After a 10 week recovery period, performance was reassessed on the supination task for 10 sessions with at least 50 trials each session, with this data being used for ‘After Injury’ point in all analyses. Animals continued training on the task for an additional 6 weeks, as detailed below. Experimenters were blinded to group throughout assessment.

### Radial nerve injury induction

All subjects underwent complete transection of the radial nerve proximal to the elbow followed by tubular repair in the trained right forelimb, as previously described^9^. Animals were deeply anesthetized with ketamine hydrochloride (80 mg/kg, IP) and xylazine (10 mg/kg, IP), and given supplemental doses as needed to maintain areflexia. A small incision was made proximal to the elbow in the right forelimb, and the radial nerve carefully isolated, exposed, and completely transected with micro-scissors. Immediately following transection, the proximal and distal stumps of the nerve were sutured 1 mm inside the opposite ends of a 6 mm saline filled polyurethane tube (Micro-Renathane 0.095″ I.D 0.066″ O.D., Braintree Scientific, Inc., Braintree, MA), resulting in a 4 mm gap between nerve stumps. The skin incision was sutured and treated with antibiotic ointment. All animals were given a single injection of enrofloxacin (7.5 mg/kg, IP) and sustained release buprenorphine (1.2 mg/kg, SC) immediately following surgery.

### Vagus nerve cuff implant

Nine weeks after nerve injury, vagus nerve stimulating cuffs were implanted in all treated animals as previously described^12,14–16^. Careful dissection of the neck exposed the left cervical vagus nerve. The nerve was isolated and placed in a bipolar cuff electrode, which was attached to a connector anchored to the skull. After VNS implant surgery, all animals were administered a single injection of enrofloxacin (10 mg/kg) and buprenorphine (0.03 mg/kg). Animals then remained in their home cage for 1 week, after which animals were randomized and underwent tactile or motor rehabilitation, as appropriate for their group.

### Tactile rehabilitation and VNS delivery

In the appropriate groups, sessions of tactile rehabilitation occurred once daily, 4 days per week, with each session lasting approximately 1.5 h, as previously described^6,7^. Each session consisted of delivery of mechanical stimulation of the right (injured) forepaw with a variety of stimuli, including a 10 g von Frey filament (North Coast Medical, Gilroy, CA), a paintbrush (Kiss Products, Port Washington, NY), a 4 mm-diameter copper rod (Everbilt, Atlanta, GA), a surgical spear (Surgical Weck Cell Spear, Beaver-Visitec International, Waltham, MA), and puffs of air delivered with a handheld bulb (Innovo Medical, Stafford, TX). Mechanical stimuli were delivered to the dorsal or ventral surface of the forepaw, as specified for each group. Individual stimuli were presented in blocks of 10 with at least 10 s between each delivery, with a total of 200 mechanical stimuli delivered each session. Each tactile stimulus was applied for approximately 1 s. Rats in the VNS groups received nerve stimulation triggered by a button press to coincide with delivery of each mechanical stimulus. Each VNS pairing consisted of a 500 ms train of pulses at 30 Hz, and each biphasic pulse was 0.8 mA in amplitude and 100 µs in pulse duration.

### Motor rehabilitation and VNS delivery

In the appropriate groups, sessions of motor rehabilitative training occurred in two 30 min sessions per day, 5 days per week, for six weeks, as previously described^12^. Each session consisted of freely performing the supination task. Rats in the Motor Rehab + VNS group received stimulation paired with successful trials during first five weeks of motor rehabilitation. The software monitoring the rotary encoder sent a trigger signal to the isolated pulse stimulator to administer VNS immediately when the rotary encoder crossed the adaptively scaled turn angle threshold. VNS parameters were equivalent to those used for tactile rehabilitation in Experiment 1 and in previous studies^5,12^. No VNS was delivered on the final week to assess effects lasting after the cessation of stimulation, which is presented as ‘After Therapy’ in the figures. All rats in the Motor Rehab group were similarly connected to the stimulator, but no stimulation was delivered during training.

### Statistical analysis and data availability

Statistical analysis was performed with MATLAB software. Forelimb withdrawal threshold data and motor performance data were normally distributed (Kolmogrov-Smirnov test, all p > 0.05). Paired t-tests were used to determine differences before and after nerve injury, and two-way repeated measures ANOVA was used to analyze the effect of time and treatment over therapy. Motor function data did not meet criteria for sphericity (Fig. 2a, Mauchly’s test, p < 0.05), so a Greenhouse–Geisser correction was applied. All data is reported as mean ± standard error of the mean (SEM) in the text and figures. All data is available upon request from the authors.

## Results

### Experiment 1: VNS paired with tactile rehabilitation fails to improve somatosensory recovery

**Figure 1:**
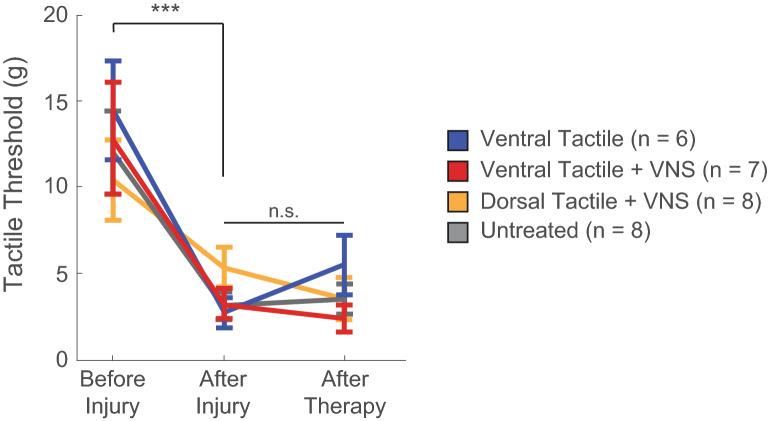
VNS paired with tactile rehabilitation fails to improve forelimb withdrawal thresholds. Radial nerve injury produces lasting allodynia in the forelimb, as demonstrated by sustained reductions in forelimb withdrawal thresholds. VNS paired with tactile therapy, consisting of mechanical stimulation of either the dorsal (Dorsal Tactile + VNS, n = 8) or ventral (Ventral Tactile + VNS, n = 7) surface of the forepaw, fails to improve withdrawal thresholds compared to tactile therapy without VNS (Ventral Tactile, n = 6) or no therapy (Untreated, n = 8). Data presented as mean ± SEM. *** denotes p < 0.001 at the indicated timepoints; n.s. denotes not significant group effect.

Previous studies show that pairing short bursts of VNS with tactile rehabilitation enhances recovery of somatosensory function in a model of nerve injury with chronic hyposensitivity^6,7^. We tested whether this implementation would also restore somatosensory function in a model of nerve injury that produces allodynia^9^. Rats underwent transection and repair of the radial nerve proximal to the elbow. As expected, radial nerve injury produced long-lasting allodynia in the ventral surface of the forelimb as measured by mechanical withdrawal thresholds (Fig. 1; Before Injury v. After injury, Paired t-test, t(32) = 6.44, p = 2.97 × 10^–7^).

All rats then received 6 weeks of tactile rehabilitation, consisting of mechanical stimulation of the ventral or dorsal surface of the forepaw, either with or without trains of VNS coinciding with mechanical stimulation. A repeated measures ANOVA comparing these interventions from after injury to after therapy revealed no significant effects of time or VNS therapy (Fig. 1; After Injury v. After Therapy; Two-way repeated measures ANOVA, Effect of time: F[1,27] = 2.34, p = 0.13; Effect of treatment: F[1,27] = 0.51, p = 0.48). Unlike results observed in models of hyposensation, these findings indicate that VNS therapy does not improve recovery of forelimb somatosensory function in a model of neuropathic pain.

### Experiment 2: VNS paired with motor rehabilitation fails to improve motor recovery

**Figure 2:**
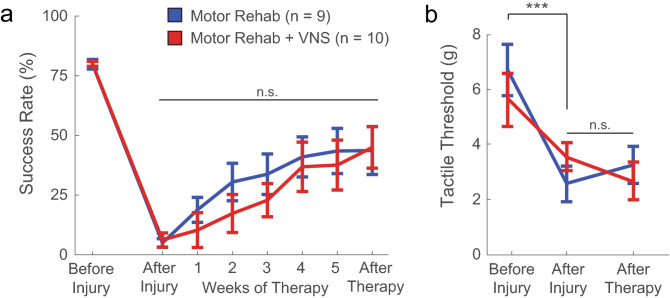
VNS paired with motor rehabilitation fails to improve recovery of skilled forelimb function. (**a**) Radial nerve injury results in chronic forelimb weakness, indicated by a sustained reduction in success rate on a skilled forelimb motor task. VNS paired with rehabilitative training (Motor Rehab + VNS, n = 8) fails to improve recovery of motor function compared to equivalent rehabilitative training without VNS (Motor Rehab, n = 8). (**b**) Additionally, VNS paired with motor training also fails to improve recovery of forelimb withdrawal thresholds. Data presented as mean ± SEM. *** denotes p < 0.001 at the indicated timepoints; n.s. denotes not significant group effect.

Pairing VNS with motor training improves forelimb strength after injury to the median and ulnar nerves^5^. We sought to determine if this strategy would also increase recovery of forelimb motor function after radial nerve injury. To do so, a separate cohort of animals was trained on a skilled motor task that required subjects to reach, grasp, and supinate their forelimb. Prior to injury, animals in both groups demonstrated similar levels of proficiency on the task. As expected, radial nerve injury significantly impaired motor performance 10 weeks after injury in both groups (Fig. 2a; Before Injury v. After injury; Paired t-test; Motor Rehab, t(8) = 29.94, p = 2.02 × 10^–9^, Motor Rehab + VNS, t(9) = 25.29, p = 1.15 × 10^–9^).

Rats then underwent 5 weeks of rehabilitative training with or without VNS delivered to coincide with forelimb movements. Motor function improved in both groups over the course of rehabilitative training (Fig. 2a; Two-way repeated measures ANOVA with Greenhouse–Geisser correction, Effect of time: F[6, 96] = 8.31, p = 1.52 × 10^–4^). Moreover, no differences in performance were observed between groups (Fig. 2a; Effect of treatment: F [1, 16] = 0.12, p = 0.73). These findings indicate that radial nerve injury produces lasting impairments in forelimb motor function and that VNS paired with training does not improve recovery of motor function.

Confirming our initial findings, radial nerve injury causes allodynia in the ventral surface of the forepaw in both groups (Fig. 2b; Before Injury v. After injury, Paired t-test, t(18) = 4.09, p = 6.76 × 10^–4^). No differences in mechanical threshold were observed across time or group, indicating that VNS paired with motor training does not confer benefits in somatosensory function (Fig. 2b; Two-way repeated measures ANOVA, Effect of time: F[2, 28]  = 0.6, p = 0.55, Effect of treatment: F[1, 14] = 0.007, p = 0.93). Overall, these results suggest that VNS therapy does not promote recovery of forelimb motor or somatosensory function after radial nerve injury.

## Discussion

In this study, we examined the effectiveness of VNS therapy on motor and somatosensory recovery in a model of nerve injury that produces neuropathic pain in the forepaw. Unlike the benefits observed in models of chronic hyposensitivity in the forepaw, we report that VNS paired with various forms of rehabilitative training fails to improve recovery of skilled motor function or reduce mechanical hypersensitivity resulting from radial nerve damage. Below, we discuss these results in context and explore possible interpretations of the current findings as they relate to the application of VNS therapy for this and other conditions.

**Figure 3:**
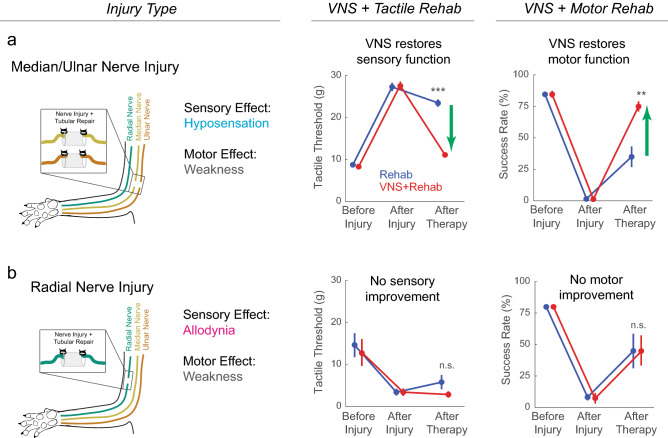
Comparison of VNS-dependent recovery in hypersensitive and hyposensitive models of nerve injury. (**a**) In previous studies, damage to the median and ulnar nerves causes somatosensory hyposensitivity and weakness in the forelimb. Pairing VNS with tactile rehabilitation significantly improves elevated sensory thresholds, restoring them to normal levels. Data from^6^. Additionally, VNS paired with motor rehabilitation significantly improves forelimb weakness compared to equivalent training without VNS. Data from^5^. (**b**) In the present study, injury to the radial nerve in the forelimb causes allodynia and weakness. Pairing VNS with tactile rehabilitation fails to restores somatosensory function. Moreover, VNS paired with motor rehabilitation fails to improve recovery forelimb strength compared to equivalent training without stimulation. The absence of VNS-dependent benefits in either somatosensory and motor recovery after radial nerve injury likely arise due to the chronic pain induced by the injury.

Previous studies demonstrate that pairing VNS with tactile rehabilitation improves recovery of somatosensory function after injury to the median and ulnar nerves in the forelimb^5–7^. We hypothesized that a similar approach may improve somatosensory recovery after radial nerve injury. However, unlike the benefits observed after median/ulnar nerve injury, VNS paired with training did not yield improvements in somatosensory function after radial nerve injury. This discrepancy likely arises from the distinct somatosensory consequences of these forms of injury: median/ulnar nerve injury produces long-lasting hyposensation in the ventral surface of the forepaw, and VNS therapy reduces sensory thresholds to normal levels (Fig. 3a). VNS is hypothesized to promote recovery by strengthening spared or restored connectivity in networks engaged by the paired rehabilitation^5,12,15,17^. Consequently, the VNS paired with tactile rehabilitation serves to strengthen the hyposensitive sensory circuits and restore normal function. Alternatively, radial nerve injury produces hypersensation, or allodynia, in the ventral surface of the forepaw and VNS does not increase sensory thresholds to normal levels (Fig. 3b).

Building on the notion that hypersensitivity produced by radial nerve injury may underlie the absence of recovery with VNS paired with ventral tactile stimulation, we sought to explore whether an alternative implementation of therapy could reduce this exaggerated response. Previous studies in the context of tinnitus, a disorder arising from hyperexcitability in auditory networks, show that pairing VNS with a range of sensory stimuli that produce hypoexcitable responses consequently reduces hyperexcitable responses to stimuli not paired with VNS^18^. We applied a congruent approach in a separate cohort of rats, in which VNS was paired with mechanical stimulation of the dorsal surface of the forepaw, which is denervated by radial nerve injury, rather than the hypersensitive ventral surface of the forepaw. This approach also failed to reduce exaggerated forelimb withdrawal responses, indicating that this implementation of VNS pairing was unable to reverse hypersensitivity. Together, these findings indicate that pairing VNS with tactile rehabilitation does not improve somatosensory function after nerve injury that produces pain.

A number of previous studies demonstrate that VNS therapy reliably enhances recovery of forelimb motor function in models of neurological injuries ranging from median/ulnar nerve injury to stroke^5–8,12–16,19^. In contrast, we observed the VNS paired with rehabilitative training failed to improve recovery of motor function compared to rehabilitative training without stimulation after radial nerve injury. Unlike the absence of VNS-dependent improvement in somatosensory recovery, this cannot be explained by functional differences between the models, as both median/ulnar injury and radial nerve injury produce forelimb weakness (Fig. 3). Moreover, it is unlikely that differences in the muscles denervated by median/ulnar nerve injury or radial nerve injury can explain the absence of motor recovery, since VNS therapy improves forelimb recovery in models of spinal cord injury and stroke that broadly impact muscles in the forelimb. Considering the totality of results, we are left to conclude that the emergence of neuropathic pain likely underlies the failure of VNS therapy to improve recovery of either motor or sensory function.

The converging actions of pain and VNS on the noradrenergic locus coeruleus (LC) may account for the absence of VNS-dependent enhancement of recovery. VNS drives rapid, phasic activation of the LC and consequent release of norepinephrine^20,21^. This engagement of the LC is required for the effects of VNS on the central nervous system^22,23^. A number of studies indicate an inverted-U relationship between LC activation and VNS-dependent effects, such that moderate levels of activity enhance plasticity and recovery, whereas higher levels of activity fail to enhance plasticity or recovery^13,20,24–27^. This inverted-U relationship is important in that higher degrees of activation do not simply fail to produce greater effects as would occur with a sigmoidal relationship, but rather paradoxically occlude the effects resulting from moderate activation^26^. Independent of the actions of VNS, painful stimuli also increase neural firing rates in the LC^28–31^. Thus, additive actions of pain and VNS on LC activity may account for absence of VNS-dependent benefits. In the absence of pain, VNS drives LC activity to a range that facilitates recovery, resulting in the consistent improvements in motor and somatosensory function observed with VNS therapy. Alternatively, in the context of pain, VNS drives activity in the LC, but the basal increase in LC activity related to pain may result in LC activity exceeding the effective range and failing to promote recovery. This raises the possibility that two strategies may yet allow VNS therapy to be effective in the context of pain. First, interventions that mitigate pain and consequently reduce LC activity to baseline levels, such as gabapentin, may permit VNS therapy to promote recovery^32^. Second, alternative VNS parameters, such as lower stimulation intensities or shorter train durations, may allow tuning of LC activity to the effective range^20,27,33,34^.

As VNS paired with rehabilitation is translated to clinical use^35^, exploring the limitations of this approach is crucial in selecting patients and optimizing delivery. Here, we provide initial evidence that neuropathic pain may limit VNS-dependent enhancement of recovery. Future efforts should evaluate existing data from clinical studies to determine if neuropathic pain is predictive of response to VNS therapy. Additionally, the results provide a framework for preclinical studies to rigorously evaluate the existence of a link between neuropathic pain and occlusion of VNS-dependent effects and explore manipulations that may restore VNS efficacy. A clear understanding of the clinical characteristics, including neuropathic pain, that may impact the effectiveness of VNS is critical to the successful translation of this therapy.
